# *Teucrium polium* L: An updated review of phytochemicals and biological activities

**DOI:** 10.22038/AJP.2021.19155

**Published:** 2022

**Authors:** Seifollah Bahramikia, Parvaneh Hemmati Hassan Gavyar, Razieh Yazdanparast

**Affiliations:** 1 *Department of Biology, Faculty of Science, Lorestan University, Khorramabad, Iran*; 2 *Institute of Biochemistry and Biophysics, University of Tehran, Tehran, Iran*

**Keywords:** Teucrium polium, Lamiaceae, Phytochemistry, Biological activities, Traditional medicine

## Abstract

**Objective:**

Medicinal plants and their components are potential novel sources for developing drugs against various diseases. *Teucrium polium* L. (syn *Teucrium capitatum* L. or felty germander) from the Lamiaceae family, is widely distributed in the dry and stony places of the hills and deserts of almost all Mediterranean countries, southwestern Asia, Europe, and North Africa. Based on traditional Iranian medicine (TIM), *T. polium* is used for treating many diseases, including abdominal pain, indigestion, and type 2 diabetes.

**Materials and Methods:**

In our previous review article published in 2012 and based on 100 articles published from 1970 to 2010, the main compounds purified from *T. polium* were terpenes, terpenoids, and flavonoids with antioxidant, anticancer, anti-inflammatory, hypoglycemic, hepatoprotective, hypolipidemic, antibacterial, and antifungal activities.

**Results:**

In this article, the phytochemistry and pharmacological activities of the plant reported from 2011 to 2020 have been evaluated. Therefore, a search was done in the databases PubMed, Science Direct and Google Scholar, Scopus, and Web of Science with the terms "*T. polium*," "*T. capitatum*." and felty germander’, which included about 100 articles published since 2011 about *T. polium* pharmacological activities and isolated compounds. Most studies of this review focused on the antioxidant and antidiabetic effects of the plant

**Conclusion:**

Considering the position of *T. polium *in folk medicine, mainly as an antidiabetic agent, purification, structural and biological characterization of the active components appears essential for effective use of the plant.

## Introduction

**Figure 1:**
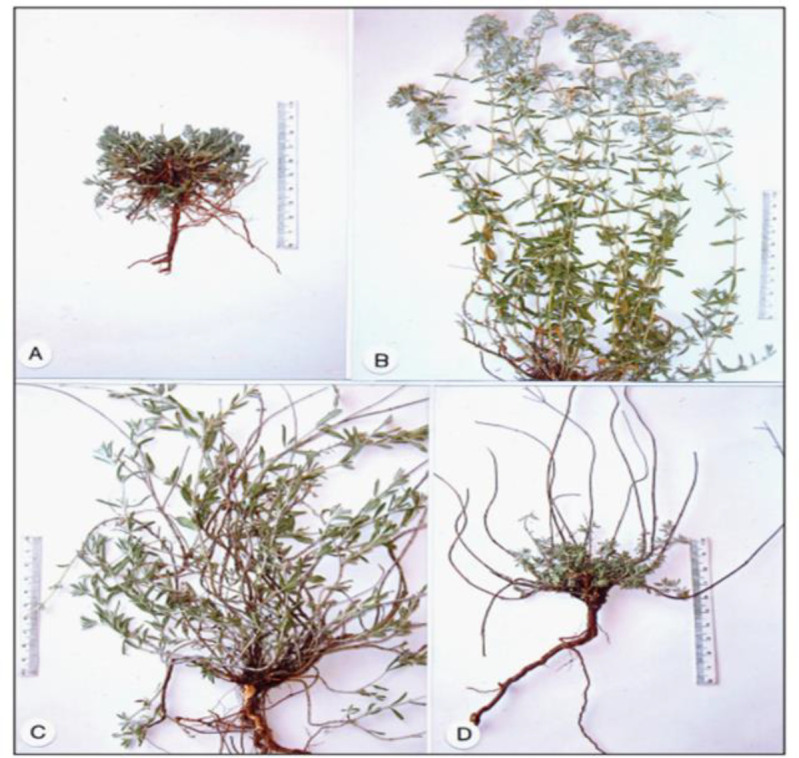
The annual biological cycle of *Teucrium polium* at various seasonal phases (herbarium material): A–winter (late January); B–spring/summer (late May/early July); C–summer (late July/middle August); D–autumn (late November)

Traditional systems of medicine provide valuable information on natural remedies. Medicinal plants, as important sources of new chemicals with potential therapeutic effects, play a significant role in discovering new drug leads. As one of the largest and most distinguished families of flowering plants, Lamiaceae has 236 genera and 6900–7200 species worldwide, with a wide range of biological activity and diverse phytochemicals (Naghibi et al., 2005 ▶; Raja, 2012 ▶). Several experimental studies on species of this family confirmed the effectiveness of some of its traditional applications. *Teucrium polium* which belongs to the family Lamiaceae, is a perennial shrub, 20*-*50 cm high, and it is widely distributed in the dry and stony places of the hills and deserts of almost all Mediterranean countries, southwestern Asia, Europe, and North Africa. Sessile, oblong, or linear leaves with a length of about 3 cm (Table 1) (Feinbrun-Dothan, 1978 ▶; Yazdanparast and Bahramikia, 2012 ▶). T. polium contains several subspecies and varieties, including aragonense, aurasiacum, capitatum cylindricum, expansum, gnaphalodes, pilosum, polium, vincentinum, yalentinum and many others (Figure 1) (El Oualidi et al., 1999 ▶). Traditionally, in the Mediterranean countries, T. polium has been used against various types of pathological conditions, such as gastrointestinal disorders, inflammations, diabetes and rheumatism (Abdollahi et al., 2003 ▶; Tariq et al., 1989 ▶). It is also used as an antibacterial, antiulcer, hypotensive, antispasmodic, anorexigenic and antipyretic agent (Autore et al., 1984; Suleiman et al., 1988 ▶; Gharaibeh et al., 1989 ▶). This plant is abundantly found in Iran and names Kalporeh. In traditional Iranian medicine (TIM), its tea is used for treating many diseases such as abdominal pain, indigestion, common cold, and urogenital diseases (Abdollahi et al., 2003 ▶). In our previous article in 2012, we mentioned many compounds mainly belonging to terpenes, terpenoids and flavonoids for which, pharmacological activities such as antioxidant, anticancer, anti-inflammatory, hypoglycemic, hepatoprotective, hypolipidemic, antibacterial and antifungal properties were reported (Bahramikia and Yazdanparast, 2012 ▶). In this article, we extend our attention to the phytochemical and pharmacological activities of the plant and its characterized constituents reported between the years 2011-2020.

**Table 1:** Scientific classification of *T. polium*

**Scientific classification**	
Kingdom:	Plantae
*Clade*:	Tracheophytes
*Clade*:	Angiosperms
*Clade*:	Eudicots
*Clade*:	Asterids
Order:	Lamiales
Family:	Lamiaceae
Genus:	*Teucrium*
Species:	*T. polium*

## Materials and Methods

In our previous review article published in 2012 based on 100 articles published from 1970 to 2010. In this article, the phytochemistry and pharmacological activities of the plant from 2011 to 2020 have been evaluated. Therefore, a search was done in the databases PubMed, Science Direct and Google Scholar, Scopus, and Web of Science with the terms "*T. polium*," "*T. capitatum*." and felty germander’, which included about 100 articles published since 2011 about *T. polium* pharmacological activities and isolated compounds. 

## Results

**Phytochemical studies**

**Components of the essential oil**

Essential oils are complex mixtures composed of terpenoid hydrocarbons, oxygenated terpenes, and sesquiterpenes. Various medical applications of these compounds include cosmetics and ingredients of medicines (Hemmati Hassan Gavyar and Amiri 2018 ▶). Up to 2011, more than 134 active substances, including diterpenoids, flavonoids, steroidal compounds, caffeic acid, and its derivatives from the aerial parts, roots and seeds of *T. polium*, have been isolated and characterized. In 2011, Bezić et al. investigated the essential oils of some *Teucrium* species including *T. polium *(Table 2). They indicated that β-caryophyllene (52%), germacrene D (8.7%), and limonene (5.9%) are major constituents of this plant. At the same time, Vahdani et al. (2011) ▶ stated that p-cymene )8.20%), limonene (37.70%), and 2,4 di-tetra-butylphenol (10.81%) are important compounds in *T. polium*. Djabou et al. (2013) ▶ indicated that the essential oil of* T.* *polium *is rich in α-pinene (33.2%), α-thujene (8.1%), and terpinen-4-ol (6.6%). The results of Hussain et al. (2013) ▶ study introduced ledene oxide (II)(20.47%), linalyl acetate (11.16%), and β-eudesmol (11.59%) as important constituents of *T.* *polium*. In another study, the main components of *T. polium* were α-cardinal (46.2%), caryophyllene oxide (25.9%), α-muurolol epi (8.1%), cadalene (3.7%), and longiverbenone (2.9%) (Khani and Heydarian, 2014 ▶). Essid et al. (2015) ▶ reported that carvacrol (56.06%), β-caryophyllene (7.68%), and α-pinene (5.02%) are the main compound of *T. polium.* Major compounds of the essential oil of *T. poliu*m were 11-acetoxyeudesman-4-a-ol (26.3%), α-bisabolol (24.6%), and β-caryophyllene (908%) in another study (Sayyad, and Farahmandfar 2017 ▶). According to the study by Othman et al. (2017) ▶, β-pinene (35.97%) and α-pinene (13.32%) were the major components of *T.* *polium*. Masoudi (2018) ▶ showed that the stem of *T.* *polium* is rich in α- muurolol (25.02%), α- cadinol (15.72%), and β-cayophyllene (10.68%). Also, the authors indicated that leaf of the plant is abundant in α-muurolol (20.03%), α- cardinal (8.11%) and β-cayophyllene (10.11%) and major components of root were α-muurolol (19.53%), α- cardinal (13.01%) and β-cayophyllene (10.46%). Two subsp of *T. polium* were studied by El Atki et al. (2019) ▶. Analysis of essential oils in their study revealed that *T. polium* subsp. *aurum* is rich in caryophyllene (19.13%), followed by γ-muurolene (13.02%), τ-cadinol (11.01%), α-gurjunene (9.2%), rosifoliol (8.79%), 3-carene (7.04%) and the main compound of *T. polium* subsp. *polium* was 3-carene (16.49%) followed by γ-muurolene (14.03%), α-pinene (9.94%), α-phellandrene (6.93%), and caryophyllene (7.51%). Fitsiou and Pappa (2019) ▶ found that the main compounds of essential oil of* T.* *polium* ssp* capitatum* were carvacrol (10.1%), caryophyllene (9.8%), and torreyol (7.6%). In chemical analysis of essential oil extraction from the aerial part of *T. polium*, lycopersene (26.00%), dodecane (14.78%), 1,5-di methyl decahydro naphthalene (9.27%), and tridecane (7.39%) were identiﬁed as the main components (Table 2) (Ebadollahi and Taghinezhad, 2019 ▶).

**Table 2:** Volatile oil compounds isolated from various parts of *T. polium*

NO	Compound name	Plant part	References
1	β-caryophyllene	Aerial parts, Leaf, Stem, Root	Bezić et al. 2011 ▶; Essid et al. 2015 ▶; Sayyad and Farahmandfar 2017 ▶; Masoudi 2018 ▶
2	Germacrene D	Aerial parts	
3	Limonene	Aerial parts	Bezić et al. 2011 ▶; Vahdani et al. 2011 ▶
4	p-cymene	Aerial parts	
5	2,4di-tetr-Butylph	Aerial parts	
6	α-Pinene	Aerial parts	Djabou et al. 2013 ▶; El Atki et al. 2019 ▶; Othman et al. 2017 ▶
7	α-Thujene	Aerial parts	
8	Terpinen-4-ol	Aerial parts	
9	Ledeneoxide	-	
10	Linalyl acetate	-	
11	β-Eudesmol	Leaf, Stem, Root	Hussain et al. 2013 ▶; Masoudi 2018 ▶
12	α-Cadinol	Aerial parts	
13	Caryophyllene oxide	Aerial parts	
14	α-Muurolol epi	Aerial parts	
15	Cadalene	Aerial parts	
16	Longiverbenone	Aerial parts	
17	Carvacrol	Aerial parts	Essid et al, 2015 ▶; Fitsiou et al. 2019 ▶
18	α-Apenine	Aerial parts	
19	11-Acetoxyeudesman-4-a-ol	Aerial parts	
20	α-Bisabolol	Aerial parts	
21	β-Pinene	Aerial parts	
22	α- Muurolol	Leaf, Stem, root	
23	Caryophyllene	Aerial parts	El Atki, et al. 2019 ▶; Fitsiou, et al. 2019 ▶
24	γ-Muurolene	Aerial parts	
25	τ-Cadinol	Aerial parts	
26	α-Gurjunene	Aerial parts	
27	Rosifoliol	Aerial parts	
28	3-Carene	Aerial parts	
29	α-Phellandrene	Aerial parts	
30	Torreyol	Aerial parts	
31	Lycopersene	Aerial parts	
32	Dodecane	Aerial parts	
33	1,5-di methyl decahydro naphthalene	Aerial parts	
34	Tridecane	Aerial parts	

**Other compounds**

*Nuclear magnetic resonance* spectroscopy (*NMR*) and mass spectrometry (*MS*) data from polar xtracts of *T. polium* showed the presence of phenylpropanoid glycosides verbascoside and poliumoside, the ﬂavones apigenin and its derivatives, and two methoxyﬂavones (Table 3) (Goulas et al., 2012 ▶). Also, at the same time, 1D and 2D NMR experiments and MS spectral analyses of methanol extract obtained from the aerial parts of *T. polium* led to the structural elucidation of ten compounds including poliumoside B, poliumoside, 8-O-acetylharpagid, teucardosid, lutenolin 7-O-rutinoside, lutenolin 7-O-neohesperidoside, lutenolin 7-O-glucoside, lutenolin 4ʹ-O-glucoside, teulamifin B, and teusalvin C (De Marino et al., 2012 ▶). In another study, analysis of CH_2_Cl_2_/MeOH extract of the aerial parts of the plant led to the identification of sixteen compounds including four sesquiterpenes 4 β,5α-epoxy-7 α H-germacr-10(14)-en-6 β -ol-1-one, 4 β,5 α -epoxy-7 α H-germacr-10 (14)-en,1b-hydroperoxyl,6 β -ol, 4 β,5 β -epoxy-7 α H-germacr-10(14)-en,1 β -hydroperoxyl,6 β -ol and 4 α,5bepoxy-7 α H-germacr-10(14)-en,1 β -hydroperoxyl,6 α -ol, together with seven known sesquiterpenes, one known iridoid glycoside, two known ﬂavonoids, and one known phenylpropanoid glycoside (Elmasri et al., 2014 ▶). For the first time, Boghrati et al. (2016) ▶ isolated four compounds, including two phenylpropanoid glycosides (verbascoside and poliumoside) and two flavonoids (jaranol and isorhoifolin) of *T. polium* var. *gnaphalodes*. In a study by Venditti et al. (2017) ▶, phytochemical analysis of *T. polium* L showed twelve compounds namely teucrasiatin (1), 20-O-acetyl teucrasiatin (2), verbascoside (3), apigenin (4), luteolin (5), acacetin (6), apigenin7-O- β –glucoside (7), cirisimaritin (8), phytol (9) oleanolicacid (10), maslinicacid (11) and pheophrorbidea (12). In another study, the presence of apigenin in *T. polium* has been reported in 2017 by Venditti.

**Table 3:** Flavonoid and other compounds isolated from various parts of *T. polium*

NO	Compound name	Plant part	References
1	Verbascoside	Aerial parts, Stem and Leaves	Goulas et al. 2012 ▶; Boghrati et al. 2016 ▶; Venditti et al. 2017 ▶
2	Poliumoside	Aerial parts	Goulas et al. 2012 ▶; Boghrati et al. 2016 ▶
3	5,3ʹ,4ʹtrihydroxy-3,7-dimethoxyﬂavone	Aerial parts	Goulas et al. 2012 ▶; De Marino et al. 2012 ▶
4	5,4ʹ-dihydroxy3,7-dimethoxyﬂavone (kumatakenin)	Aerial parts	
5	Apigenin 7-O-rutinoside	Aerial parts	
6	Apigenin 7-O-glucoside	Aerial parts	
7	Apigenin 4 ʹ O-glucoside	Aerial parts	
8	Apigenin	Aerial parts, Stem and Leaves	Goulas et al. 2012 ▶; Venditti et al. 2017 ▶; Venditti. 2017 ▶
9	Poliumoside B		
10	8-O-acetylharpagid		
11	Teucardosid		
12	Luteolin 7-O-rutinoside		
13	Luteolin 7-O-neohesperidoside		
14	Luteolin 7-O-glucoside		
15	Luteolin 4ʹ-O-glucoside		
16	Teulamifin B		
17	Teusalvin C		
18	4 β,5 α-epoxy-7 α H-germacr 10(14)-en-6 β -ol-1-one	Aerial parts	
19	4 β,5 α -epoxy-7 α H-germacr-10 (14)-en,1b-hydroperoxyl,6 β -ol,	Aerial parts	
20	4 β,5 β -epoxy-7 α H-germacr-10(14)-en,1 β -hydroperoxyl,6 β -ol	Aerial parts	
21	4 α,5bepoxy-7 α H-germacr-10(14)-en,1 β -hydroperoxyl,6 α -ol,	Aerial parts	
22	Jaranol	Aerial parts	
23	Isorhoifolin	Aerial parts	
24	Teucrasiatin	Stem and Leaves	
25	20-O-acetyl teucrasiatin	Stem and Leaves	
26	Luteolin	Stem and Leaves	
27	Acacetin	Stem and Leaves	
28	Apigenin7-O- β –glucoside	Stem and Leaves	
29	Cirisimaritin	Stem and Leaves	
30	Phytol	Stem and Leaves	
31	Oleanolic Acid	Stem and Leaves	
32	Maslinic Acid	Stem and Leaves	
33	Pheophrorbidea	Stem and Leaves	

**Ethnobotanical studies and traditional medicine use**

In different parts of the world, different parts of this plant are used in various forms, alone or combined with other plants in traditional medicine. It has been reported that a cup of infusion of the leaves of *T. polium* after a meal is recommended because of its antidiarrheal, hypnotic, antiparasitic, antifungal, and antitussive actions. Also, the usage is recommended to treat diabetes mellitus, rheumatoid arthritis, paranasal sinusitis, bloating, menorrheal discharge, wound disinfection, gingivitis, tonsillitis, acne, itching, dyspepsia, and amenorrhea (Miikaili et al., 2012 ▶). *T. polium* L. is traditionally used in the town of Elazığ, Turkey, for high cholesterol, cold, and ﬂu. *T. polium* L. extract was shown to induce hypoglycemic, antipyretic, and intestinal motility activities. The leaves are used for diabetes, kidney and liver diseases, stomach and intestinal pain, diabetes and hemorrhoids Hayta et al. (2014) ▶. The results of a research conducted in Edremit Bay showed that *T.* *polium* was extensively used for commercial purposes. Infusion of flowering and branches of *T. polium *L. is used in traditional medicine to treat diabetes and kidney stones (O.Ad. (Oral administration) by drinking one teacup two times a day for one week (Polat and Satıl, 2012 ▶). In the markets of Mashhad, Iran, *T. polium* are used by the traditional medicine for antacid, indigestion, diabetes, treatment of colic and diarrhea (Amiri and Joharchi, 2013). Reports indicated that aqueous extract of* T. polium *with tail flick test showed antinociceptive activity. It's possible mechanism is inhibiting release of acid arachidonic and synthesis of prostaglandins and effect on opioid system binding to pain receptors, affecting ligand-sensitive channels and decreasing sodium entrance (Bahmani et al., 2014 ▶). In another study, Nasab and Khosravi (2014) ▶ reported that fumes from burning *Descurainia* *sophia* seeds combined with *T*. *polium* flowers heal earaches and ear infections. Also, flower extract / plant powder of *T*. *polium* are used to treat chickenpox, ear infections, ear pain, abdominal ache, diarrhea in infants, acne and skin blemishes. Sadeghi et al. (2014) ▶ reported that flowers and branches of *T. polium* L. used in traditional medicine to treat antipyretic, insect, snake, and scorpionbite; also, it is used for wound healing, stomach ache, abdominal pain, ﬂatulency, emesis, stomach acidiﬁcation, hypertension, toothache, diabetes, and hyperlipidemia and administered as a sedative. Ethnopharmacological use of them was reported for gastrointestinal disorders, common cold, and fever, and as an antioxidant, antispasmodic, hypoglycaemic, and anti-inﬂammatory agent (Eissa et al., 2014 ▶). Two years later, Ali-Shtayeh et al. (2016) ▶ reported that aerial part of* T. polium *in ethno-veterinary is used to treat diarrhea, colic, bleeding, scabies, and flatulence. *T. polium *as a medical treatment in Bordj Bou Arerridj region, Northeast Algeria, is used as infusion and powder to treat diabetes and migraine. Ouelbani et al. (2016) ▶ reported that this plant is used in traditional medicine for the treatment of wounds, coagulants, chills, fever, and digestive system problems. Also, it is used for treatment of wound, hemorrhoids, weakness, chills, fever, and pinworms and as an anti-inflammatory, astringent, detergent, febrifuge (paludisme), anti-hyperglycemia, disinfectant, stomachic, hypotensive, coagulant, vermifuge, and antidiabetic agen (Miara et al., 2019 ▶).

**Biological activities**

In our previous article in 2012, based on 100 articles published from 1970 to 2010, several pharmacological activities, including antioxidant, anticancer, anti-inflammatory, hypoglycemic, hepatoprotective, hypolipidemic, antibacterial, and antifungal effect of different extracts and compounds isolated from *T. polium* were reported. In this study, all the biological effects of the plant reported from 2011 to 2020 are shown (Table 4).

**Table 4:** Biological activities of *T. polium*

Biological activities	References
Antioxidant activities	Krishnaiah et al. 2011 ▶; Goulas et al. 2012 ▶; De Marino et al. 2012 ▶; Khaled-Khodja et al. 2014 ▶; Vladimir-Knežević et al. 2014; Boghrati et al. 2016 ▶; Sayyad and Farahmandfar 2017 ▶; El Atki et al. 2019 ▶; El Atki et al. 2020 ▶; Asadi and Farahmandfar 2020 ▶
Cytotoxic and anticancer	Stankovic et al. 2011 ▶; Movahed et al. 2014 ▶; Dağ et al. 2014 ▶; Essid et al, 2015 ▶; Kristanc and Kreft 2016 ▶; Rahmouni et al. 2017 ▶; Al-Qahdi et al., 2019 ▶, Vilas-Boas et al., 2020 ▶
Antibacterial, antiviral and antifungal activities	Bezić et al. 2011 ▶; Vahdani et al. 2011 ▶; Djabou et al. 2013 ▶; Khaled-Khodja et al. 2014 ▶; Khani and Heydarian 2014 ▶; Othman et al. 2017 ▶; Ravan, et al. 2019 ▶; Ebadollahi and Taghinezhad 2019 ▶
Memory enhancement	Williams et al. 2011 ▶; Hasanein and Shahidi 2012 ▶; Ali et al. 2013 ▶; Knežević et al. 2014; Ahmadian-Attar et al. 2015 ▶; Mousavi et al. 2015 ▶; Simonyan and Chavushyan 2016 ▶ Lobbens et al. 2017 ▶
Anti-Ischemic and antiseizure effects	Khoshnood-Mansoorkhani et al. 2010 ▶; Mahmoudabady et al. 2018 ▶
Anti-inflammatory activity	
Hypolipidaemic effects	Niazmand et al, 2017 ▶; Safaeian et al. 2018 ▶
Cardiovascular effects	Niazmand et al. 2011 ▶; Sheikhbahaei et al. 2018 ▶; Mahmoudabady et al. 2014 ▶; Nor et al. 2019 ▶
Hepatotoxicity	Fiorentino et al. 2011 ▶; Forouzandeh et al. 2013 ▶; Rafieian-Kopaei et al. 2014 ▶; Jadeja, et al. 2014 ▶; Jadeja et al. 2014 ▶; Baali et al. 2016 ▶; Lin et al. 2019 ▶; Pour et al. 2019 ▶
Wound healing activity	Alizadeh et al. 2011 ▶; Hosseinkhani et al. 2017 ▶; Meguellati et al. 2019 ▶
Effect on sexual hormones	Khadige et al. 2016 ▶; Salimnejad et al. 2017 ▶
Pain reducing effect	Khadige et al. 2016 ▶; Uritu et al. 2018 ▶

**Antioxidant activities**

Different studies have shown that overproduction of free radicals and oxidative stress cause various diseases, including cancer, cataracts, cardiovascular disease, immune system decline, and brain dysfunction. Thus, using plants such as *T*. *polium*, which are rich in antioxidant compounds, is an important strategy against these diseases (Hemmati Hassan Gavyar and Amiri, 2018 ▶). In a review article by Krishnaiah et al. (2011) ▶, several compounds including rutin, apigenin, 3', 6-dimethoxy apigenin, and 4', 7-dimethoxyapigenin from the methanolic solvent of *T. polium* (aerial parts) were reported as the main components with high antioxidant ability as assessed by 2, 2-diphenyl-1-picrylhydrazyl (DPPH) radical scavenging and β – carotene bleaching assays ( IC_50_=20.1±1.7 µg/ml and 25.8±1.2 mm, respectively). Goulas et al. (2012) ▶, using High performance *liquid chromatography*-solid phase extraction-*nuclear magnetic resonance* )HPLC-SPE-NMR( and HPLC-DPPH techniques, indicated that phenylpropanoid glycosides purified from the polar extracts of *T*. *Polium *are responsible for antioxidant activity (66-80%). Different methods examined the effects of extraction by various solvents on the antioxidant activity of leaves of *T. polium*. The results showed that among the various extracts, the n-butanol extract has higher antioxidant power, likely due to the presence of compounds such as ﬂavonoids, iridoids, and phenylethanoids (De Marino et al., 2012 ▶). In another study, antioxidant activities of methanolic extracts of some Lamiaceae species, including *T. polium*, were studied by Khaled-Khodja et al. (2014) ▶. Their research showed that among examined plants, *Mentha pulegium* and *T*. *polium* had high antioxidant activity. Also, *T. polium* had the highest total phenolics and total ﬂavonoids contents compared to other plants. Antioxidant activities of the ethanolic extracts of selected Lamiaceae species, including *T.* *polium*, were investigated by Vladimir-Knežević et al., 2014 ▶. Their findings showed that *T.* *polium *extract is a powerful antioxidant (IC_50_=5.90±0.12 µg/ml). Verbascoside, poliumoside, jaranol, and isorhoifolin were the four compounds isolated from *T.* *polium* var. gnaphalodes; Jaranol showed the highest tyrosinase inhibitory activity, and poliumoside had the highest antioxidant activity as assessed by fluorescence recovery after photobleaching )FRAP( (14.32 mmol/g) and DPPH radical scavenging ) IC_50_ =0.042 µg/ml) assays compared to other compounds (Boghrati et al., 2016 ▶). Sayyad and Farahmandfar (2017) ▶ reported that essential oil of *T. polium* can be useful to oxidative stability of the canola oils. Rahmouni et al. (2017) ▶ reported that *T. polium* has protective effect on hematological and some biochemical parameters against carbon tetrachloride (CCl4) induced toxicity in rats. In another study, antioxidant activity and total phenolic and flavonoid contents of methanol, ethanol, water, and ethyl acetate extracts of *T. polium* were investigated by El Atki et al. (2019) ▶ who showed that in both methods (DPPH radical scavenging and FRAP), the methanolic extract had the highest antioxidant activity (lowest IC_50_). Also, the total phenolic and flavonoid contents of this extract were higher than water, and ethyl acetate extracts. In addition, the highest total antioxidant capacity was related to water extract. El Atki et al. (2020) ▶ identified that the essential oils of *T. polium* subsp. *aurum* have a higher antioxidant power than *T. polium* subsp. *polium*, in both methods DPPH radical scavenging and FRAP. In addition, results showed that in the total antioxidant capacity method,* T*. *polium* subsp. *aureum* had a signiﬁcant activity (3308.27 mg equivalent to ascorbic acid/g of EO). Recently, it was reported that *T. polium* extract could be used as a natural antioxidant for the stability and safety of canola oil during frying. Tocopherols and phenolic compounds in the extract are probably responsible for this feature (Asadi and Farahmandfar, 2020 ▶). 

**Antidiabetic activities**

*T* *.* *polium *has been long recommended in Iranian folk medicine for its anti-diabetic activities. In a study by Kasabri et al. (2011) ▶, *in vivo* and *in vitro*, antihyperglycemic effects of five selected indigenous plants from Jordan used in traditional medicine were investigated. *In vitro* model results demonstrated that *T. polium* did not have appreciable anti-amylase or anti-glucosidase effectiveness. In addition, *T. polium* aqueous extracts did not evoke any substantial reduction in the overall glycemic excursion in the treated animals. Each *T. polium*-supplemented group had a significant decrease (p<0.05) in cornstarch-induced acute hyperglycemia 45 min post intragastric starch administration. They concluded that other modes of action could explain their substantial antihyperglycemic activities in starch-treated rats (Kasabri et al., 2011 ▶). In a study by Tatar et al. (2012) ▶, the effects of *T. polium* aerial parts extracts on oral glucose tolerance tests and pancreas histology in streptozocin-induced diabetic rats, were investigated. The histopathological investigation, along with the biochemical evaluations, indicated that treatment of diabetic rats with *T. polium* resulted in the regeneration of the pancreatic islets and reduction of the severity of streptozotocin-diabetic pancreases. The authors concluded that the extract of the aerial parts of *T. polium* probably stimulates pancreas repair and may be clinically beneficial as an agent to restore or maintain pancreas tissue after injury. Mousavi et al. (2012) ▶ investigated the effects of *T. polium* ethyl acetate extract on serum, liver, and muscle triglyceride content of sucrose-induced insulin resistance in rats. The treatment of rats with *T. polium* ethyl acetate extract resulted in a dose-dependent reduction in serum, liver, and muscle triglyceride (TG) and liver glycogen content levels and serum insulin. They concluded that these effects might be attributed, in part, to the hypolipidemic, hepatoprotective, and antioxidant activity of *T. polium* flavonoids. In a study performed by Mousavi et al. (2015) ▶, the beneficial effects of *T. polium* and metformin on diabetes-induced memory impairments and brain tissue oxidative damage in rats were evaluated. Results indicated that treatment with decoctions of *T. polium* for six weeks relieves the deleterious effects of diabetes on learning and memory. Phenolic-rich *T. polium* reduces oxidative damage to the hippocampus and cerebral cortex synapses, thus correcting learning and memory deficits in diabetes patients.

Regarding the significant effects of *T. polium* on β-cell regeneration and insulin secretion in animal models of type 1 diabetes, Tabatabaie and Yazdanparast (2017) ▶ investigated the molecular mechanism involved in the β-cell regeneration. Their results indicated that the antidiabetic effect of *T. polium* is strongly mediated via the antioxidant defense system and the Pdx1 expression in the JNK pathway of the streptozotocin (STZ)-induced diabetic rats pancreas. Recently, in a study by Amrae et al. (2020) ▶, the effects of the different fractions of *T. polium *on the aldose reductase enzyme (AR) activity as a strategy to reduce retinopathy were investigated. Results indicated that all fractions were found to inhibit lens AR activity. Among the different fractions and crude extract, the ethyl acetate fraction had the highest AR inhibitory activity (IC_50_= 3.67 μg/ml). In addition, the results showed noncompetitive inhibition of AR by the ethyl acetate fraction of *T. polium*.

**Anticancer activities**

In a study by Stankovic et al. (2011) ▶, antiproliferative and proapoptotic activity of methanolic extracts from different *Teucrium* species and its effect on the prooxidant/antioxidant status in HCT-116 cells were investigated. MTT assay indicated that all species, including *T. polium*, significantly reduced cell viability in a dose-dependent manner, with very low IC_50_ values. Among all species, the methanol extracts from *T. polium* had a moderate cytotoxic effect after 24 hr (IC_50_= 77.83±0.4 µg/ml) and 72 hr (IC_50_= 253.39±1.61 µg/ml) of exposure. Also, the methanolic extracts of *T. polium* had a remarkable effect on superoxide anion radical (O_2_^−^) and nitrite (NO_2_^−^) production in HCT-116 cell line after 24 and 72 hr of exposure. Their results indicated that these effects are attributed to the phenolic and flavonoids compound contents of the plants. Anticancer activity of *T. polium* on hepatocellular carcinogenic rats was studied by Movahed et al. (2014) ▶. The results showed that *T.* *polium* suppresses liver cancer development and this may be due to the high levels of flavonoids and antioxidant compounds in the plant. 

**Antibacterial, antiviral, antifungal, and antileishmanial activities**

Essential oils isolated from *Teucrium* species, including *T. polium*, were investigated for antiphytoviral activity; this species showed moderate reduced Cucumber Mosaic Virus (CMV) infections (41.4%) (Bezić et al., 2011 ▶). In another study, Vahdani et al. (2011) ▶ showed that *T.* *polium* has mild antimicrobial activity on microorganisms. Djabou et al. (2013) ▶ identified that Corsican *Teucrium* essential oils have the potential to be used as food preservatives and to prevent the growth of nosocomial bacteria. In another study, the antibacterial effect of methanolic extracts of some Lamiaceae species, including *T. polium* against *Escherichia coli* and *Staphylococcus aureus*, has been tested by agar disk diffusion and micro-dilution assays. Results showed that *T. polium* extract has low antibacterial activity (Khaled-Khodja et al., 2014 ▶). The essential oils of two species, *T. polium* subsp. *aurum* and *T. polium* subsp. *polium* were tested for antibacterial effects against two nosocomial bacteria, and the results showed that both species had high antibacterial properties, especially against *Tribolium* * castaneum* and Callosobruchus* maculatus*. Also, the results from their study suggest that sesquiterpene-rich essential oils from *T. polium* subsp. *capitatum* (L.) had insecticidal activity and could be used as a potential control agent for stored-product insects (Khani and Heydarian, 2014 ▶). Antimicrobial effects of essential oil, ethanol, and aqueous extracts of *T. polium* L. were tested against 13 microorganisms. The results indicated that essential oil possessed the highest antimicrobial activity and was most effective against *Proteus mirabilis*, *S. aureus*, and *Citrobacter freundei*. Also, essential oils and ethanolic extracts showed high antifungal power against *Microsporum* *canis*, *Scopulariopsis* *brevicaulis*, and *Trichophyton* *rubrum* (inhibition percentage 18.94 to 100%). None of the samples had antifungal activity against *Aspergillus* *fumigatus* (Othman et al., 2017 ▶). Two years later, Ravan et al. (2019) ▶ reported that essential oil from *T. polium* could be used as a potential agent to control aphid. They showed that exposure to sublethal concentrations of essential oil caused a reduction in the intrinsic rate of natural increase (r_m_ value) because it decreased the adult female longevity and fertility of surviving aphids. Simultaneously, results from a study by Ebadollahi and Taghinezhad (2019) ▶ on essential oils of *T. polium* showed high insecticidal efﬁciency against red ﬂour beetle, and toxicity was increased with increasing exposure time and the amount of sublethal concentration.

In another study, essential oil from *T. polium *was evaluated for antileishmanial activity. The results illustrated that essential oil has potent inhibitory activity against two promastigote forms* L. major *and* L. infantum, *even its antileishmanial activity was higher than amphotericin B (control positive); the cytotoxicity on macrophage cells was low (Essid et al., 2015 ▶).

**Effect on memory enhancement**

In a review study, Williams et al. (2011) ▶ reported that extract from the aerial parts of *T. polium* is anti-amnesic *in vivo* and inhibits Acetylcholinesterase (AChE) *in vitro*. In another study, it was stated that this plant possesses a protective effect against memory impairment in diabetes (Hasanein and Shahidi 2012 ▶). Ali et al. (2013) ▶ indicated that aerial parts of *T. polium* improve mental performance and focus. AChE inhibitory activities of the ethanolic extracts of 26 medicinal plants of the Lamiaceae family, including *T. polium, *were investigated by Vladimir-Knežević et al. (2014) ▶. The results of this study showed that some of these species, including *T. polium *at 1 mg/ml indicated strong inhibitory activity against AChE. Also, Ahmadian-Attar et al. (2015) reported that in traditional medicine, the decoction of the aerial part of this plant is prescribed orally due to its anti-depressant properties. Mousavi et al. (2015) ▶ reported that the hydroalcoholic extract of *T. polium* inhibit diabetes-induced memory deficits in rats. In another study, it was reported that *T. polium* reduced  ovariectomized (OVX)-induced neurodegenerative alterations in entorhinal cortex-hippocamp circuitry and facilitated neuronal survival by modulating neurotransmitters' activity and network plasticity (Simonyan and Chavushyan 2016 ▶). Lobbens et al. (2017) ▶ reported that in Anatolia, this plant is used in traditional medicine to enhance memory. 

**Anti-ischemic and antiseizure effects**

In a research by Khoshnood-Mansoorkhani et al. (2010), protective effects of *T. polium*ethanolic aqueous extracts and related fractions on seizures induced by pentylenetetrazole (PTZ)and maximal electroshock stimulation (MES) have been investigated. Their results showed that aqueous extract of *T. polium *and a related n-butanol fraction (ED_50_=12.6 mg/kg body weight) have antiseizure effects comparing to control groups and ethanolic extract. Authors believed that high levels of flavonoids in the aqueous extract may be the reason for this difference. 

Regarding the deleterious effect of oxidative stress on myocardial ischemia-reperfusion (I/R), Mahmoudabady et al. (2018) ▶ investigated the effects of the *T. polium* on I/R injuries in the isolated rat heart. Their results indicated that pretreatment with *T. polium* increased thiol (SH) groups, *superoxide dismutase* (SOD), and catalase (CAT) activities but decreased the lactate dehydrogenase (LDH), creatine kinase (CK) activity, and TBARS level; therefore, it can be concluded that this plant has a cardioprotective effect against oxidative stress during I/R injury. 

**Cardiovascular effects**

Cardiovascular effects of *T. polium* extract in rabbits were investigated by Niazmand et al. (2011) ▶. Their study showed that aqueous-ethanol extract had no effect on heart rate but showed a positive inotropic on the heart. In addition, the extract of *T. polium* indicated hypotensive effect. The reason for these observations is the inotropic effect of the extract. Results from a study about the synergic effect of *T. polium* and tranilast on human umbilical vein endothelial cells (HUVECs) by Sheikhbahaei et al., 2018 ▶ showed that this plant in a dose- and time-dependent manner, alone or in combination reduced the viability of HUVECs. Mahmoudabady et al. (2014) ▶ indicated that *T. polium* extract could help to prevent high blood pressure induced by Angiotensin II (Ang II) pathway activation. In another study, *in vitro* antiatherothrombotic effects of extracts from three species, including *T. polium*, were studied. The data showed prolonged coagulation time in a concentration-dependent manner following administration of aqueous extract and polysaccharide extract from *T. polium* implying a potential antithrombotic property for this plant (Nor et al., 2019 ▶).

**Hepatoprotective activity**

In a study by Fiorentino et al. (2011) ▶, the hepatoprotective activity of seven neo-clerodanes (teupolins VI-XII) and eleven known compounds isolated and purified from a polar extract of *T. polium* leaves against the human hepatoblastoma cancer cell line HepG2 was evaluated. Data from a study by Forouzandeh et al. (2013) ▶, suggest that *T. polium* aqueous-ethanol extract has a protective effect on acetaminophen-induced hepatotoxicity in mice because their findings showed a decrease in the serum liver enzyme activities (alanine aminotransferase (ALT), aspartate aminotransferase (AST), and alkaline phosphatase (ALP)) and bilirubin concentrations. Moreover, the liver morphology and histopathology findings confirmed the protective activity of this extract against the acetaminophen-induced liver damage as shown by the reversal of centrilobular necrosis, fatty changes (steatosis) and scattered lymphocytes infiltrate in hepatic parenchyma by *T*. *polium* administration. Also, Rafieian-Kopaei et al. (2014) ▶ reported that *T. polium* has a protective effect on hepatotoxicity. At the same time, Jadeja et al. (2014) ▶ reported that alkaloid from *T. polium* extract has hepatotoxicity. Jadeja et al. (2014) ▶ reported that ethyl acetate fraction of *T. polium* could be used for the treatment of nonalcoholic steatohepatitis. Protective activity of total polyphenols from *Genista quadriﬂora* *Munby* and *T. polium* *geyrii* Maire in acetaminophen-induced hepatotoxicity in rats, was investigated by Baali et al. (2016) ▶. Their study results showed that polyphenolic extracts of *T. polium* and *G. quadriﬂora* had hepatoprotective activity and reduced transaminase leakage. At the same time, in a review study, Pour et al. (2019) ▶ reported that *T. polium* is useful for the hardness of the spleen, splenitis, black jaundice, and dropsy (ascites).

**Wound healing activity**

Alizadeh et al. (2011) ▶ indicated that honey of *T. polium* could assist wound healing and tensile strength in rat skin wounds. Also, Hosseinkhani et al. (2017) ▶, based on the belief of traditional Persian medicine, showed that *T. polium* aerial part could be used for various wounds. The results of studies conducted by Meguellati et al. (2019) ▶ identified that the extract of this plant has the property of healing skin wounds in rats, the use of callus obtained from the aerial parts of *T. polium* improved the wound after 11 days which by far exceeded the threshold marked by the reference.

**Effect on sexual hormones**

Khadige et al. (2016) ▶ in a triple-blind placebo-controlled clinical trial, investigated the effect of *T. polium *on reducing menstrual bleeding. The result of their study showed that *T. polium* significantly decreases duration and amount of menstrual bleeding in the 1st and the 2nd menstruation cycles after treatment. In another study, the effect of *T. Polium* extract administration on spermatogenesis and testicular structure in diabetic rats induced with streptozotocin was investigated. Results showed that the hydroalcoholic extract of *T. polium* has a protective effect on diabetes-induced testicular damage and serum testosterone concentration. This effect was related to antioxidant and antidiabetic properties of the hydroalcoholic extract of *T*. *polium* (Salimnejad et al., 2017 ▶).

**Pain reducing effect**

Khadige et al. (2016) ▶ showed that *T. polium* was as effective in reducing the pain severity in primary dysmenorrhea as mefenamic acid. In a review article, Uritu et al. (2018) ▶ reported that *T. polium* might be eﬀective in some type of pain, for example, visceral pain (Abdollahi et al., 2003 ▶), menstrual cycle pain, pain-related behavior in the diabetic rat formalin test (100 or 200 mg/kg body weight) (Baluchnejadmojarad et al., 2005 ▶). In addition, the ethanolic extract of the plant in dose of 500 mg/kg bodyweight inhibited carrageenan-induced inﬂammation and reduced granuloma formation (Tariq et al., 1989 ▶).

**Hypolipidaemic and anti-inflammatory effects**

The effects of a polyherbal mixture, including *T. polium*, were tested on biochemical parameters in diabetic rats. The results showed that this polyherbal mixture has beneficial effects on blood glucose and lipid profile (Niazmand et al., 2017 ▶). The finding of the study by Safaeian et al. (2018) ▶ indicated that various fractions derived from hydroalcoholic extract of *T. polium* had a strong antihyperlipidemic effect, but chloroform fraction had the highest hypolipidemic activity in a dose‑dependent manner. In a study on the anti-inflammatory effects of the plant, the essential oil from the aerial parts of *T. polium *ssp* capitatum *showed anti-inﬂammatory activity, being able to inhibit LPS-induced NO production (Fitsiou and Pappa, 2019 ▶).

**Cytotoxicity**

Dağ et al. (2014) ▶ indicated that *T. polium* has a potential hepatotoxic effect; however, physiological changes during pregnancy and postpartum periods may increase the severity of such toxicity that should be considered in the differential diagnosis. Kristen and Kreft in 2016 ▶ reported that many plant species of the genus *Teucrium* including *T. polium*, cause moderate liver damage in rodents. Furan-containing diterpenoids are toxic components of the extract, which after about a month can lead to cholestatic hepatitis. Al-Qahdi et al. (2019) ▶ showed that women should avoid taking *T. polium* plants during pregnancy because the plant can have very toxic effects on the early stage of the embryo. Lin et al. (2019) ▶ reported that *T. polium* from Greece induced liver injury. Recently, it was reported that *T. polium* might lead to liver injury (often with a cholestatic signature). The toxicity mechanism is related to furano neoclerodane diterpenoids, teucrin A and teuchmaedryn A, with too highly reactive epoxides considered as inducers of hepatocyte apoptosis (Vilas-Boas et al., 2020 ▶).

## Discussion

Our *T. polium* literature search covering the years 2011 up to 2020 indicated that most of the studies focused on the antidiabetic and antioxidant activities of the various extracts of the plant. Due to the role of oxidative stress in many diseases on one hand and the high antidiabetic and antioxidant potency of the plants, on the other hand, it is predicted that the *T. polium* extracts and/or elucidated components of the extracts could be beneficial for the treatment of a wide range of diseases, pending further investigation to eliminate the cytotoxicity, if any, of the crude or partially purified fractions of *T. polium.*

## Conflicts of interest

The authors have declared that there is no conflict of interest.
